# Strategies to expand peptide functionality through hybridisation with a small molecule component

**DOI:** 10.1039/d0cb00167h

**Published:** 2020-12-08

**Authors:** Yuteng Wu, Jack Williams, Ewen D. D. Calder, Louise J. Walport

**Affiliations:** Protein-Protein Interaction Laboratory, The Francis Crick Institute London UK Louise.walport@crick.ac.uk; Department of Chemistry, Molecular Sciences Research Hub, Imperial College London London UK

## Abstract

Combining different compound classes gives molecular hybrids that can offer access to novel chemical space and unique properties. Peptides provide ideal starting points for such molecular hybrids, which can be easily modified with a variety of molecular entities. The addition of small molecules can improve the potency, stability and cell permeability of therapeutically relevant peptides. Furthermore, they are often applied to create peptide-based tools in chemical biology. In this review, we discuss general methods that allow the discovery of this compound class and highlight key examples of peptide–small molecule hybrids categorised by the application and function of the small molecule entity.

## Abstract 2

This review details discovery strategies and established applications of peptide-small molecule hybrids, classified by the added functionality imparted by the small molecule component.

## Graphical abstract

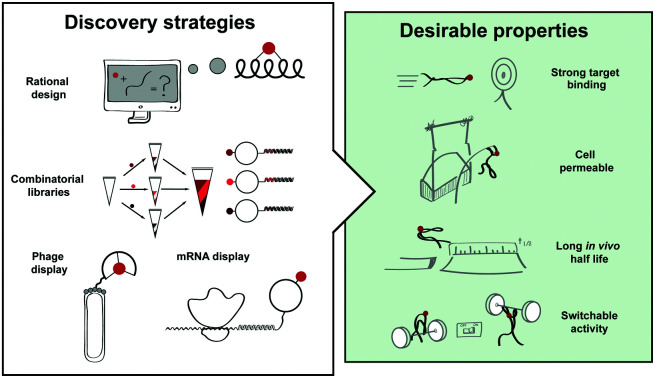

## Introduction

Peptides are a promising class of drug molecule that are also particularly useful as tools in chemical biology. With a size range between small molecules and biologics, they occupy a Goldilocks region, that when optimised, have the potential to adopt some of the favourable properties from each: the high potency and selectivity of biologics, and the higher bioavailability and cellular permeability associated with smaller molecules.^1,2^ This unique place in chemical space, makes them particularly applicable to modulating targets previously thought of as undruggable, such as protein–protein interactions.^3–5^

Despite this great potential, achieving the optimal balance of favourable properties is challenging, and as yet their development and use in the clinic or *in vivo* systems has been limited.^2,6^ Smaller peptides, which may have favourable pharmacokinetic properties, often have inferior binding affinity to biologics. By contrast, larger peptides with enhanced potency, tend to have reduced stability and cell permeability as a result of their size, limiting them to extracellular targets.

Strategies to optimise the drug-like qualities of peptides are continually being developed.^7–10^ Their inherent modularity means their structure and sequence can be easily tweaked to enhance their efficacy. Conjugation to natural biomolecules such as fatty acids, steroids or sugars is a well-established technique to modulate peptide properties.^11–16^ Small molecule components have also been incorporated into peptides to generate ‘peptide–small molecule hybrids’. Depending on the intended function, conjugation of the small molecule motif can occur directly on an amino acid side chain, through the amide backbone or, for cyclic peptides, through the cyclisation linkage itself.^17–19^ These additions modulate the properties of the parent peptide, producing hybrids that show significant potential in addressing the limitations of peptides as therapeutic agents or tools in chemical biology.

The aim of this review is to illustrate the potential of peptide–small molecule hybrids in both the development of pharmaceuticals and as tools for basic research. Following an overview of screening strategies by which peptide–small molecule hybrids can be identified, we detail prominent hybrid examples classified by the function of the small molecule component. First, we describe examples where the hybrid exhibits enhanced binding affinity attributed to the incorporation of the small molecule component. In the second section, we discuss the use of small molecule tags to improve the pharmacokinetics of peptides through prolonging their *in vivo* half-life or improving their cell permeability. Finally, we highlight examples where the hybrids have applications as tools in chemical biology, primarily as switchable molecules and photoaffinity probes. There are various standard peptide–small molecule hybrids that are not covered in this review. In particular, the well-established use of peptides to improve the efficacy of a known small molecule or drug, in the form of a peptide–drug conjugate is not covered and the interested reader is directed to one of the many excellent reviews on this topic.^20–24^

## 1. Discovery strategies

When developing hybrid molecules, the conceptually simplest approach is to begin with a small molecule or peptide which is known to bind to the target protein. These validated ligands can provide the starting point for the rational design of peptide hybrid molecules with enhanced properties, often with assistance from structural data. At present this approach is the most widely used, and examples of this approach are discussed below. However, validated binders and high-resolution crystal structures are not always available. A more generalisable approach requiring no prior knowledge of the target is therefore to screen vast small molecule–peptide hybrid libraries. This allows the *de novo* discovery of lead compounds for almost any target of choice.

**Fig. 1:**
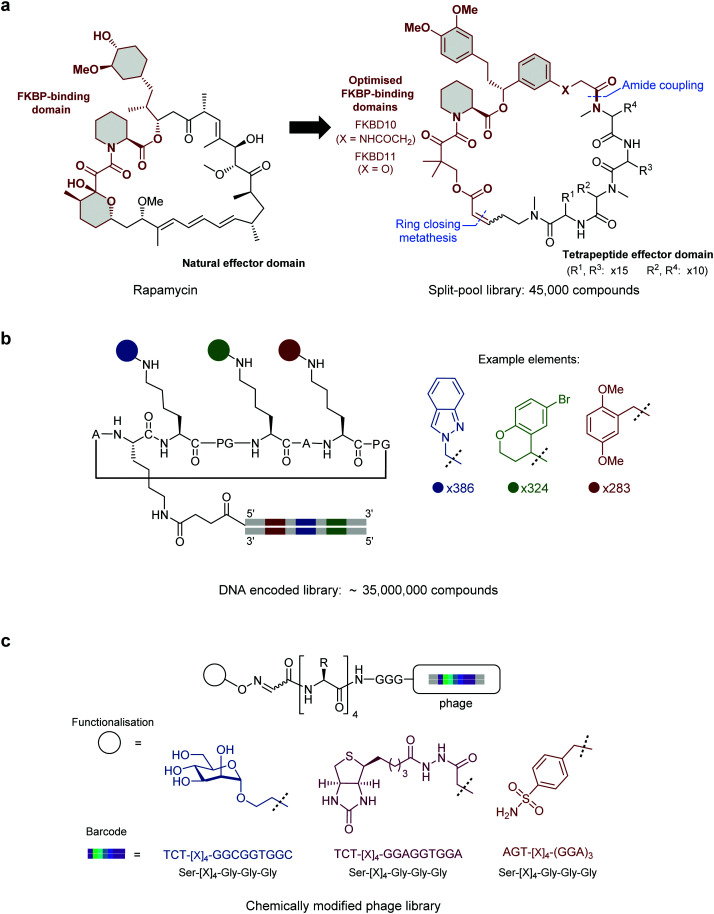
Strategies for constructing vast small molecule–peptide hybrid libraries. (a) Split-pool library of Rapamycin-like hybrid macrocycles comprised of an optimised FKBP-binding domain fused with a tetrapeptide effector domain. For **A15-34-8**: X = NHCOCH_2_, R^1^ = Phenylalanine, R^2^ = *N*-methyl-d-Phenylalanine, R^3^ = Proline, R^4^ = *N*-methyl-Leucine. (b) DNA encoded library bearing a structurally-defined cyclic peptide scaffold with three encoded small molecule diversification sites. (c) Phage-displayed peptide library functionalised with mannose, biotin or sulphonamide each encoded by a silent barcode. [X]_4_ = randomised (NNK)_4_ library.

An established method for generating diverse hybrid libraries is the split-and-pool technique, where libraries of up to 10^7^ molecules can be produced through combinatorial chemistry.^25^ As these libraries are constructed synthetically, highly diverse building blocks can be incorporated into library members. In a notable example of this approach, Liu and co-workers reported a ring-closing metathesis-based strategy to generate a library of 45 000 rapamycin-like hybrid peptide macrocycles.^26^ Rapamycin and FK506 are macrocyclic natural products which share an FK506-binding protein (FKBP) binding motif as well as an effector domain which binds mTOR (Rapamycin) or calcineurin (FK506). By generating ternary complexes between FKBP12 and their target protein, Rapamycin and FK506 allosterically block substrates from reaching the active site of mTOR and calcineurin respectively. In a successful attempt to hijack this interesting mode of action, Liu and co-workers individually fused two optimised FKBP-binding domains (**FKBD10**, **FKBD11**, Fig. 1a) with a combinatorial library of solid-phase synthesised tetrapeptides intended to replace the effector domain and so alter their selectivity. Screening of the library in human cells identified a potent inhibitor (**A15-34-8**, IC_50_ = 426 nM) of human equilibrative nucleoside transporter 1 (hENT1). Further optimisation of the tetrapeptide sequence in the hybrid improved the potency by 85.2-fold.

DNA-encoded libraries are an extension of the split-and-pool approach which can generate and screen libraries of up to 10^12^ compounds.^27^ In a DNA-encoded library, each combinatorial element is assigned a unique nucleotide barcode which is attached in tandem with each synthetic step. After affinity panning of the library against an immobilised protein target, molecules that bind can be identified by sequencing of the attached unique DNA barcode. This allows for pooled screening of much larger libraries than can be achieved with traditional tag-free combinatorial libraries, where library members must be screened individually. In an impressive application of this technique Scheuermann, Neri and co-workers reported a strategy to display multiple diverse chemical elements, each encoded by a DNA barcode, on a structurally-defined macrocyclic scaffold which adopts an antiparallel beta-sheet conformation (Fig. 1b).^28^ Various chemical elements were incorporated through three diversity sites resulting in the construction of an impressive 35 393 112 compound library. Subsequent selections with this library yielded specific binders against eight proteins, including carbonic anhydrase IX, calmodulin and prostate-specific antigen. By exchanging the DNA-barcode for fluorescein and modifying the scaffold to add a fourth functionalised diversity site, the identified hybrid peptide binders were converted to chemical probes. For example, a phenyl azide moiety was fused to a calmodulin binder identified from the library to yield a compound that preferentially photo-crosslinked to calmodulin in the presence of human serum albumin.

By contrast to the above methods, phage display and mRNA display use DNA to directly produce peptide libraries by translation, removing the need for numerous synthetic steps. In each case, the translated peptides remain associated with their encoding DNA, allowing the generation of libraries that are orders of magnitude larger than those produced by DNA-encoded synthesis.^1,29,30^

In phage display libraries, small molecule components can easily be incorporated as the cyclising linker or as a post-translational modification. This allows for the construction and screening of enormous hybrid libraries.^31^

One of the first applications producing peptide hybrids using phage display was the pioneering work of Christian Heinis and co-workers in developing bicyclic peptide hybrids. By incorporating three cysteine residues into the phage displayed peptides, linear peptides were bi-cyclised with a set of tri-electrophilic linkers for screening against targets of interest.^31^ Varying ring size,^32^ linker,^33^ and number of cyclising cysteines^34^ can enhance diversity in the resulting peptide macrocycles. Such libraries have been used successfully to identify binders of a wide range of proteins including plasma kallikrein,^31,35^ urokinase,^36^ β-catenin,^37^ and coagulation factor XII.^38^ Additionally, these libraries can be used to develop chemical probes with the incorporation of a photoswitching motif, as discussed in Section 2.3. Recently, the approach has been modified to introduce protease pressure during selection, with the aim of identifying therapeutic peptides that are resistant to gastrointestinal proteases after oral administration.^39^ The strategy was successful in identifying a peptide inhibitor of coagulation Factor XIa and an antagonist for the interleukin-23 receptor, which were proteolytically stable in the gastrointestinal tract and more suitable for oral administration.

Subsequently, Derda and co-workers expanded the phage display toolbox by using phage libraries incorporating “silent barcodes” (unique combinations of redundant codons) to identify peptides that had been post-translationally modified with small molecule fragments.^40^ The barcodes enable mixing of libraries with different fragment additions which can be deconvoluted following DNA sequencing. In a proof-of-concept study, phage-displayed peptide libraries were individually functionalised with one of three small molecule modifications (mannose, biotin, or sulphonamide, Fig. 1c) and the libraries combined. Hits were identified for concanavalin A, streptavidin, or carbonic anhydrase (known binders of these functionalities) through affinity panning with the pooled libraries. This approach has since been used with particular effect to target carbohydrate-binding proteins.^41,42^ In a recent extension of this approach, the post-translational incorporation of a 1,3-diketone greatly expanded the functionality that could be incorporated in the library.^43^ Having installed the diketone on their “silent barcode” phage-displayed libraries, Derda and co-workers then diversified by using a Knorr pyrazole synthesis with a wide range of hydrazine containing reagents. Using this methodology, they were able to incorporate fluorophores, lipids, PEG chains, metal chelators and groups known to bind certain proteins. Specifically, by installing a sulphonamide, known to bind carbonic anhydrase IX, they use their method to find a nanomolar potency peptide–small molecule hybrid. This “silent barcode” approach has the potential to be applied more widely in both phage and mRNA display technologies and could allow the screening of much larger libraries with a wide variety of post-translational modifications.

Another prominent screening method for the development of peptide–small molecule hybrids is mRNA display and in particular the random non-standard peptide integrated discovery (RaPID) system. The RaPID system can incorporate a wide range of non-canonical amino acids at multiple sites in the final peptide library with relative ease. Successful examples include *N*-alkyl, β- or d-amino acids, as well as more exotic hybrid-like amino acids such as carboranylalanine.^44–46^

Such an approach also allows rational incorporation of designed small molecule warheads, to customise libraries for a specific target. In an early example, Suga and co-workers produced RaPID libraries containing an ε-*N*-trifluoroacetyl group, a weak mechanism-based inhibitor of SIRT2, to search for improved inhibitors.^47^ The study resulted in the identification of two potent inhibitors of SIRT2 (*K*_D_ and IC_50_ in the single digit nanomolar region), which importantly displayed impressive isoform selectivity for SIRT2 over other isoforms SIRT1 and SIRT3. In a more recent example, Payne and co-workers employed a similar strategy to screen for cyclic peptide binders for chemokine CCL11, with peptides possessing key sulfo-Tyrosine (sTyr) residues.^48^ Four high affinity binders were identified (*K*_d_ < 30 nM) that inhibited CCL11 activation of its cognate receptor CCR3, with the sulfated residue found to be vital for achieving inhibition.

The use of foldamers to mimic peptide secondary structures is a well-established field,^49–51^ but only recently have the two classes of molecule been combined to form hybrid species. In a prominent example, Gellman and co-workers designed peptides that contain β-amino acid residues in addition to standard α-residues. When optimised, the replacement of α with β-amino acids can lead to improvements in proteolytic stability as well as target selectivity.^52–56^ In another example, Huc, Suga and co-workers demonstrated the ribosomal synthesis of helical aromatic foldamer–peptide hybrid molecules, with the large foldamer component, based on quinoline and pyridine monomers, incorporated at the peptide N-terminus.^57^ In follow up work, the methodology was extended to incorporate foldamers within and at the C-terminus of the peptide sequence.^58^ Though not yet validated in a protein selection, incorporation of such large non-peptidic entities may give rise to desirable properties such as greater water solubility, protease resistance and cell permeability.

As the field of peptide–small molecule hybrids grows, the ability to develop these molecules without relying on known peptide binding sequences will only increase in importance. The expansion of techniques that allow the construction and screening of vast (10^6^ to 10^15^ members) libraries of diverse hybrid molecules is already beginning to show great promise in the delivery of this class of compound. Further expansion of these approaches will be key to the development of this field.

## 2. Functionalities imparted by small molecule component

The addition of small molecules can impart a variety of different functions to the resulting hybrid. In the context of enhancing the drug-like properties of peptides and their functionality as chemical tools, we have chosen to focus on three main areas: (1) enhanced target binding, (2) improved pharmacokinetics and (3) switchable activity. In the following section, we discuss key examples of peptide–small molecule hybrids categorised by the application and function of the small molecule entity.

### 2.1 Binding

A major application of peptide–small molecule hybrids is to improve upon the binding affinity achieved with peptides alone. Though peptides often display greater binding affinities for their target than smaller molecules (due in part to their larger size), it is rare they can reach the potency and specificity of biologics. Conjugation of a small molecule to a validated binding peptide (linear or cyclic) can enhance affinity through additional interactions provided by the small molecule component. Alternatively, the incorporation of reactive moieties can lead to improved potency through covalent bonding with the target. Finally, pre-organising the peptide *via* a small molecule-mediated macrocyclisation can enhance ligand affinity by reducing the entropic penalty upon target binding. In these cases, the cyclisation linkage can also participate in binding by forming direct interactions with the target.

**Fig. 2:**
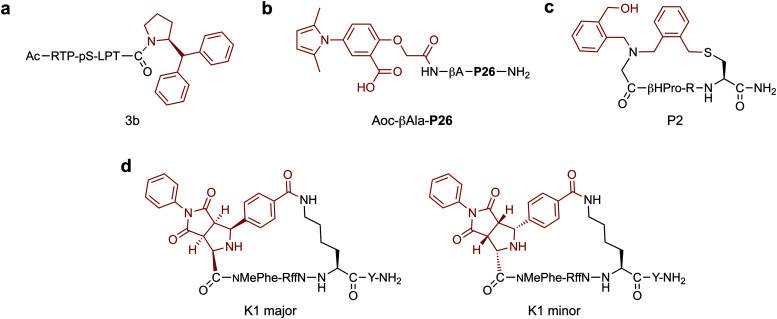
Small molecule fragments for generating high affinity hybrids. (a) Benzhydryl pyrrolidine group for targeting the fusicoccin A binding pocket in 14-3-3. pS = phosphoserine (b) *N*-aryl pyrrole moiety for interacting with the hydrophobic pocket of HIV-1 gp41. **P26** = NNYTSLIHSLIEESQNQQEKNEQELL. βA = β-Alanine (c) Nanomolar thrombin inhibitor with an exocyclic hydroxymethyl-benzyl group which induced a new hydrophobic cavity in the protein binding pocket. βHPro = l-β-Homoproline. (d) Selective MC5R binding hybrid (**K1 major**) generated by stereoselective 1,3-dipolar cycloaddition. The diastereoisomer, **K1 minor**, was unable to bind to the target. NMePhe = *N*-methyl-l-phenylalanine. f = d-phenylalanine. In each case the small molecule component is highlighted in red.

In a notable example, where improved affinity was achieved by expanding the binding interface of a validated peptide, Ottmann and co-workers rationally developed a hybrid inhibitor (**3b**, Fig. 2a) of the 14-3-3/Tau interaction.^59^ Comparison of crystal structures of 14-3-3 bound to an inhibitory Tau epitope or a small molecule natural product, Fusicoccin A (FC), led to the identification of a region of overlap between the two binding interfaces. Based on this information, peptide–small molecule derivatives were designed. By starting from the Tau pS213 phosphopeptide epitope and attempting to also probe the proximal FC binding pocket, the interaction interface was extended. In particular, addition of a C-terminal benzhydryl pyrrolidine group significantly improved 14-3-3 binding affinity (∼225-fold) by addressing the hydrophobic pocket targeted by the A-ring of FC. The work was further developed by varying the amino acid adjacent to the small molecule and further exploring substituents on the aromatic rings, which resulted in the discovery of a library of low micromolar hybrid inhibitors of the 14-3-3/Tau interaction.^60^

Apart from engaging in additional interactions with the target, small molecules are often explored to replace segments of a binding peptide. Ideally, the resulting hybrid would retain the high potency of the parent peptide whilst simultaneously imparting other favourable properties, such as improved stability. For example, Liu and co-workers investigated *N*-aryl pyrroles for developing potent HIV-1 gp41 binding hybrids with improved stability.^61^ Hybrid development started from a peptide sequence known as C34, a nanomolar potency binder, containing a short region which interacts with a deep, hydrophobic pocket on HIV-1 gp41. A truncated version of C34 (P26, 26-AAs) lacking this region was designed and coupled with a range of *N*-aryl pyrrole moieties. The *N*-substituted pyrroles were chosen based on a series of known low micromolar HIV-1 gp41 fusion inhibitors. Whilst the best hybrid (**Aoc-βAla-P26**, IC_50_ = 14.9 nM, Fig. 2b) showed around a 10-fold loss in potency when compared to the full length C34 peptide, it had a 170-fold gain when compared to the truncated peptide. Importantly, **Aoc-βAla-P26** also displayed better stability in a proteinase K digestion assay compared to C34. This highlights the ability of hybrids to combine the advantages and minimise the respective shortcomings of the two classes of compounds: loss of potency from peptide truncation was restored by conjugation of small molecules, simultaneously improving the stability of the resulting compounds.

Covalent cross-linking to the target protein represents a distinctive approach for enhancing the binding of peptides. Current strategies involve the rational incorporation of reactive motifs into validated binding sequences. For example, Hoppmann and Wang developed covalent inhibitors of MDM4 by addition of an exocyclic aryl sulfonyl fluoride group.^62^ The reactive group was attached to the side chain of an unnatural amino acid and inserted within a known MDM4 binding sequence. The peptide–small molecule hybrid was able to bind MDM4 covalently through proximity-enabled bioreactivity, resulting in a 10-fold improvement in p53/MDM4 inhibition over the unmodified peptide sequence. Similarly, Spring and co-workers utilised an alternative electrophilic warhead, a sulfotetrafluorophenyl (STP) ester group, for cross-linking to MDM2.^63^ Successful cross-linking relies on an appropriately positioned, suitably reactive amino acid. Fortunately, there are a wide range of options beyond electrophilic warheads including photoreactive groups (see Section 2.3), making this technique widely applicable. Screening methods to incorporate the reactive motifs in the starting libraries would extend this even further.

Finally, macrocyclisation is an established methodology for improving the binding affinity of peptides. Whilst this can be achieved directly through forming a head-to-tail peptide bond or a side-chain mediated disulfide bond, small molecule moieties are often used to achieve this. A notable example of such a methodology was reported by Fasan and co-workers who developed macrocyclic organo-peptide hybrids (MOrPHs).^64^ The first step involves the copper-catalyzed azide–alkyne cycloaddition (CuAAC) of an azido bearing synthetic precursor with an alkyne on the backbone of an intein-fused polypeptide. In the second step, the thioester bond at the intein junction is then intercepted by a hydrazide moiety on the synthetic precursor to simultaneously form the cyclic peptide and expel the intein protein. Variations of the technique include replacing the synthetic precursor with an oxyamino/amino-thiol trifunctional alternative,^65^ allowing catalyst-free cyclisation, and the incorporation of two cysteines in the polypeptide backbone to form bicyclic macrocycles through a disulfide bridge.^66^ In an application of the MOrPH strategy, HDM2/X binding peptides were constrained into their alpha-helical conformation by the small molecule synthetic precursor.^67^ The resulting macrocycles displayed inhibitory activity of the p53/HDM2 interaction, however the small molecule motif did not directly participate in binding. This cyclisation strategy has recently been integrated with phage display with the displayed macrocyclic libraries successfully applied to identify a high-affinity binder for streptavidin (*K*_D_ = 20 nM), and potent inhibitors of Keap 1 (*K*_D_ = 40 nM) and Sonic Hedgehog (*K*_D_ = 550 nM).^68^

Of the large variety of macrocyclisation chemistries currently available, the majority focus only on cross-links that constrain the peptide fragment of the molecule.^17–19,69^ For example, constraining the peptide through stapling can promote secondary structure formation in the form of alpha helices.^18,70^ However, in some cases, the cyclisation linkage can also contribute directly to target engagement, either by serendipity or design. In a notable example reported by Sawyer and co-workers,^71^ the hydrocarbon linkage in an alpha helical MDM2/MDMX dual inhibitor was itself found to form hydrophobic interactions on the surface of the target protein. The optimised peptide ATSP-7041 bound both MDM2 (*K*_i_ = 0.9 nM) and MDMX (*K*_i_ = 6.8 nM) with nanomolar affinities. Similar linkages have since been designed to participate in binding for other alpha helical peptides,^72,73^ as well as irregularly structured peptides.^74^ For example, apart from the structurally minimal hydrocarbon motif, Spring and co-workers demonstrated a relatively large and complex bis(triazolyl) linkage can also engage in favourable hydrophobic binding interactions with MDM2, analogous to that of the hydrocarbon cross-link.^75^

Incorporation of structurally diverse or complex linkages can generate peptide libraries with unique backbone structures, that may contribute to enhanced binding. Heinis and co-workers recently reported an efficient macrocyclisation reaction based on thiol-to-amine ligations using a set of diverse bis-electrophiles.^76^ A library of 8988 macrocycles was constructed by reacting 1284 unique tetrapeptides with seven different linkers. Subsequent screening against thrombin led to a selective nanomolar inhibitor (*K*_i_ = 42 nM). Interestingly, the most potent compound (**P2**, Fig. 2c) had undergone a second addition of the linker, connecting an exocyclic *N*-(2-(hydroxymethyl)benzyl) substituent to the macrocycle *via* the secondary amine in the backbone. A structure–activity relationship analysis showed the unforeseen hydroxymethyl-benzyl group was essential for binding, such that a shift of the hydroxymethyl group from the two to the four-position resulted in a ∼100-fold drop in potency. X-ray crystallography studies revealed a new hydrophobic cavity was created in the protein binding pocket to accommodate the hydroxymethyl-benzyl group. In a follow up study, it was found that the simultaneous substitution of l-β-homoproline with d-β-homoproline, and hydroxymethyl-benzyl with furfuryl resulted in a 5.5-fold improvement in potency over **P2**.^77^

Many cyclisation chemistries are currently available to add functionalisation in a diversity orientated fashion.^78–82^ In a recent example, Waldmann and co-workers developed a strategy to add natural product inspired small molecule structures *via* the cyclisation linkage.^83^ The structurally complex fragments were incorporated into macrocyclic hot loop-derived peptides by imine formation and subsequent stereoselective 1,3-dipolar cycloaddition using nine unique dipolarophiles. Screening against two protein–protein interactions demonstrated that the absolute configuration of the small molecule addition was essential in achieving optimal potency. In the first example, natural product like structures were cyclised with peptides derived from the iNOS hot loop to yield a series of nanomolar binders of the hSPSB2 adaptor protein. The second example utilised ARGP RFF hot spot sequences to develop selective ligands and agonists for the melanocortin 5 receptor (MC5R). Importantly, a major diastereomer (**K1 major**, Fig. 2d) was found to bind to MC5R (IC_50_ = 4.1 μM) with 7-, 5-, and 13-fold selectivity over MC1R, MC3R and MC4R respectively, whereas the minor diastereomer (**K1 minor**, Fig. 2d) did not display binding to any of the receptors. Whilst the current work focused solely on using the small molecule scaffolds to conformationally constrain the peptide part of the macrocycle, such diverse natural product-like linkages show great potential in also contributing favourable binding interactions themselves.

Many of the described peptide–small molecule hybrids successfully improve target binding affinity. In the few cases where binding affinity is only retained when comparing the hybrid to the peptide starting point, the hybrid holds other advantages such as superior stability by reducing the susceptibility to proteases. Overall, they demonstrate the potential of such hybrids in tailoring the binding affinity of therapeutic peptides. Optimal hybrid structure can be determined through rational design when a crystal structure of a peptide starting point is readily available. However, we have also highlighted a number of examples where serendipitous features of hybrid molecules have been identified, emphasising that inclusion of such hybrids in the initial screening strategy is likely to be an effective way of identifying high affinity binders.

### 2.2 Pharmacokinetics

#### 2.2.1 *In vivo* half-life

A key limitation of many therapeutic peptides *in vivo*, is their short half-lives in circulation (often *t*_1/2_ ∼ min),^84^ due to both rapid digestion and excretion. Many strategies have been developed to reduce susceptibility to proteases, including through constraining the peptide backbone, for example through cyclisation, and inclusion of non-canonical amino acids.^85^ However, the small size of peptide therapeutics means intact peptides are also excreted quickly through renal filtration (proteins <30 kDa are rapidly excreted through the glomeruli).^86,87^ A strategy to counter this and thus improve the lifetimes of peptides *in vivo* is through direct fusion to serum proteins, such as albumin, or by conjugation to fatty-acid/peptide-based tags that bind to albumin.^88–90^ Due to its large size, conjugation or binding to albumin increases the *in vivo* lifetimes of peptides by preventing renal filtration. Additionally, it has been demonstrated that binding to albumin can increase proteolytic stability, further increasing half-lives. PEGylation has also been successfully employed to increase circulation time by dramatically increasing the size of the molecule.^91,92^ However, many of these earlier strategies suffer from limitations, including, (i) reduction in *in vivo* efficacy of the peptide due to the large tag, (ii) low binding affinity to serum protein, (iii) poor solubility of resulting conjugate, (iv) difficult synthesis of the conjugate, (v) poor tissue and cell penetration and/or (vi) potential to cause an immunogenic response.^84,91,93,94^ To overcome these, strategies involving the conjugation of small molecule serum protein ligands to therapeutic peptides have been described.^84,95–100^ The resulting peptide–small molecule hybrids display significantly improved *in vivo* half-lives whilst maintaining biological activity, and do not possess the same limitations as the earlier fusion protein-, lipidation-or PEGylation-based approaches.

The earliest reported small molecule-based strategies involved conjugation of naphthalene acylsulfonamide or phosphate ester-based tags to improve *in vivo* half-lives of peptide anticoagulants.^95,96^ More recent strategies have mostly focussed on improving the *in vivo* pharmacokinetic properties of Glucagon like peptide-1 (GLP-1) analogues, such as extendin-4, which are used in the treatment of type 2 diabetes.

**Fig. 3:**
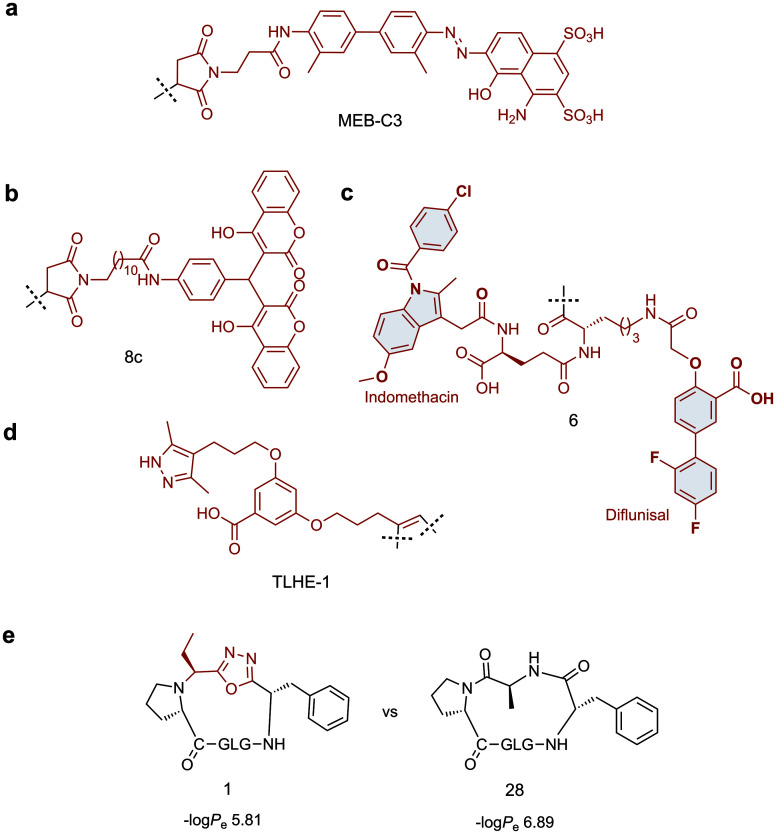
Small molecule scaffolds for prolonging *in vivo* half-life or cell permeability of peptides. (a) Stable maleimide modified version of Evans blue dye with high affinity for serum albumin. (b) Dicoumarol maleimide-based tag for enhancing the *in vivo* half-life of Glucagon like peptide-1 analogues. (c) Divalent albumin binding tag comprised of Diflunisal and Indomethacin. (d) Transthyretin-binding small molecule ligand for improving the *in vivo* half-life of Gonadotropin-releasing hormone. (e) Oxadiazole-containing cyclic peptide displaying higher passive membrane permeability compared to its homodetic counterpart. Small molecule components are highlighted in red.

Towards this goal, Chen and co-workers have developed two tag variants that are based on a maleimide-modified version of Evans blue dye (MEB), a small molecule with high affinity for serum albumin.^97^ MEB was conjugated to Cys40 of extendin-4 to give Abextide, which displayed superior bio-distribution over extendin-4 with both intravenous and subcutaneous injection. The *in vivo* half-life of Abextide (*t*_1/2_ = 36.28 ± 7.01 h) was 7-fold longer than that of extendin-4 (*t*_1/2_ = 5.16 ± 5.23 h), with concomitant increase in hypoglycaemic duration in Abextide-treated type 2 diabetic mice compared with extendin-4-treated mice (36 h and 10 h respectively). Despite the significantly improved distribution, pharmacokinetic and anti-diabetic properties of Abextide, the thiol–maleimide tag is susceptible to hydrolysis, prompting Liu *et al.* to develop a more stable MEB tag (**MEB-C3**, Fig. 3a).^98^ Conjugation of **MEB-C3** to extendin-4 gave abextide II, which displayed superior stability to hydrolysis over Abextide I. Not only was Abextide II an improvement on extendin-4, but also Albiglutide, a commercial extendin-4-albumin fusion, over which it maintains a longer hypoglycaemic state and better blood glucose control.

An alternative strategy for improving the *in vivo* half-life and efficacy of GLP-1 analogues was devised by Han *et al.* who exploited the potent binding of 4-hydroxycoumarin to human serum albumin.^99^ The approach involved conjugating a dicoumarol maleimide-based tag (**8c**, Fig. 3b) to cysteine residues in the peptide backbone of potent GLP-1 analogues. Two compounds were identified that displayed significantly improved *in vivo* half lives in rats (*t*_1/2_ = 22.07 ± 1.1 h, *t*_1/2_ = 18.78 ± 2.79 h) compared with that of extendin-4 (*t*_1/2_ = 2.82 ± 0.21 h), whilst maintaining comparable antidiabetic properties.

A novel approach involving divalent albumin binding was employed by Bech *et al.* who developed a tag comprised of two small molecule albumin binding drugs, Diflunisal and Indomethacin (**6**, Fig. 3c).^100^ When conjugated to GLP-1 analogues, the avidity of the divalent peptide–small molecule hybrid for albumin resulted in increased *in vivo* half-life over a monovalent analogue where only the Indomethacin tag was attached (*t*_1/2_ = 299 min *vs.* 55 min) as well as displaying protracted absorption into the blood after sub-cutaneous injection.

The strategies discussed so far have all employed albumin as the serum protein target for delaying renal clearance. In contrast, Penchala *et al.* developed a strategy targeting the 55 kDa homo-tetramer Transthyretin (TTR) utilising a potent, selective and reversible small molecule TTR ligand (AG10).^84^ Model peptides were conjugated to an AG10 based tag (**TLHE1**, Fig. 3d) using click chemistry. Conjugation of **TLHE1** to Gonadotropin-releasing hormone (GnRH, *t*_1/2_ = 2–6 min) dramatically improved its *in vivo* half-life giving an initial distribution phase of 12 min followed by an extended terminal phase (46 ± 3 min, 13-fold longer than GnRH *t*_1/2_). Furthermore, tag addition to a proteolytically stable analogue of GnRH (GnRH-A, *t*_1/2_ = 55 ± 11 min) increased its *in vivo* half-life more than 3-fold, with an associated improvement in *in vivo* efficacy over the un-tagged peptide. Importantly, it was demonstrated that the peptide–**TLHE1**/TTR interaction was reversible and did not interfere with the peptide binding to its target, GnRH-R.

#### 2.2.2 Cell permeability

As discussed previously, another common limitation of many peptide therapeutics is poor cell permeability. Cellular uptake mechanisms are generally not well understood, but some promising design strategies and principles to promote endocytic uptake and release are beginning to emerge.^101,102^ Cell permeability is frequently enhanced through conjugation to a cell-penetrating peptide (CPP).^103–106^ Other general approaches involve tailoring the peptide sequence and charge, or through cyclisation.^107–110^ Recently however, peptide–small molecule hybrid strategies have also been reported which show promise in promoting cell permeability of the peptide of interest.

An innovative strategy for promoting cellular uptake has been developed by Matile and co-workers, utilising the ability of constrained cyclic disulphides to pass through a lipid bilayer by dynamic covalent thiol–disulphide exchange.^111,112^ The methodology was then extended to diselenolanes, which showed improvement over dithiolanes in delivering fluorophores to the cytosol of HeLa Kyoto cells.^113^ Recently, it has been demonstrated that these diselenolanes can be used to transport a range of cargos into the cytosol, including peptides, proteins and quantum dots, suggesting that this approach may be widely applicable.^114^

Whilst the introduction of conformational constraint in the form of macrocyclisation can in itself improve membrane permeability,^115,116^ enhancement is not guaranteed. Strategic design of the cyclising moiety can promote enhanced uptake, however. In an impressive example reported by Yudin and co-workers, the addition of a 1,3,4-oxadiazole scaffold within the peptide backbone was found to improve the passive membrane permeability of cyclic peptides.^117^ The approach utilises a multicomponent cyclisation reaction between a linear peptide, an aldehyde and a (*N*-isocyanimino)triphenylphosphorane. The reaction has a broad substrate scope, tolerating various sequences and ring sizes (15-, 18-, 21- and 24-membered rings), although amino acid side chain protection is required for the ring closure step and a proline is preferred as the N-terminal residue. In a parallel artificial membrane permeability assay, the oxadiazole-containing macrocycles showed higher permeability compared with their homodetic counterparts (*e.g.* compound **1***vs.***28**, Fig. 3e). The noncanonical backbone region was shown to serve as an endocyclic control element that promotes a unique intramolecular hydrogen bond network, stabilising beta-turn motifs and facilitating passive membrane permeability. Applying this strategy to biologically relevant peptides and evaluating their uptake properties in a biological context will permit wider application of this approach.

In an example where peptide–small molecule hybrids were investigated in a biological context, Spring and co-workers found a small molecule carrier (SMoC) group incorporated through the cyclising linker can enhance the cellular uptake of a series MDM2-binding peptides.^118^ SMoCs are molecules that possess guanidinium cations linked to a lipophilic biphenyl system; they were designed by Selwood and co-workers to be a smaller and potentially more proteolytically stable mimic of guanidine-rich cell-penetrating peptides.^119^ Higher uptake for the SMoC-bearing MDM2-binding peptide was visualised by confocal microscopy, which correlated with an improvement in p53 activation in cells. However, the SMoC group was found to induce toxicity in some sequence variants in a lactate dehydrogenase leakage assay. This highlights that care should be taken when appending small molecule moieties to peptides, and that sequence optimisation to avoid unwanted toxicity may be required.

The above strategies highlight the potential of peptide–small molecule hybrids for improving the pharmacokinetic properties of therapeutic peptides. Strategies to improve *in vivo* half-life are well validated and whilst many have been exclusively applied to GLP-1 analogues, they have the potential to be applicable to other peptide therapeutics. Furthermore, it is likely that there are many more serum-binding small molecule ligands that could be employed in a similar fashion and this is a topic that warrants further investigation. Importantly, these ligands must bind reversibly to the serum protein, without compromising the function of either the carrier protein or the therapeutic peptide. Small-molecules are less likely to negatively impact peptide solubility than fusion proteins, fatty-acid chains and PEGylation, and their small size means they rarely interfere with *in vivo* peptide function. Linker length is an important factor to be considered and likely needs optimisation on a case by case basis. Small molecule strategies to improve cellular uptake are more limited and the majority of existing approaches highlighted here are not yet generalisable. With better understanding of permeability mechanisms, new methods and small molecules can be developed to overcome this challenge.

### 2.3 Chemical tools

Chemical biology probes are powerful reagents for studying the mechanistic and phenotypic roles of protein targets. The majority of these molecules comprise a section which binds the target of choice, combined with a group or molecule that imparts the additional functionality of the probe. We have chosen here to focus on switchable molecules, photoaffinity probes and chemically-triggered tools. Conjugation of small molecule dyes, such as rhodamine, is a standard method for peptide visualisation and thus will not be discussed.^120^

Incorporation of a switch into a molecule makes it possible to “turn-on”—and in some cases “turn-off”—a probe's activity in a highly spatio-temporally controlled manner. Switchable small molecules allow the study of topics such as rapid kinetic responses^121–124^ and the activation or inhibition of a protein in a specific region of a cell.^125–127^ The investigation of switchable probe molecules also opens the way for switchable drug molecules,^128–130^ and the development of pro-drugs.^131^ There are numerous switchable small molecule probe motifs and the expansion of these into peptides has yielded some fascinating results.

Photo-switchable probes represent one of the largest classes of switchable molecules. In general, the switchable part of these probes is centred around a double bond which can be isomerised by irradiation with particular wavelengths of light. Irradiation with one wavelength sets the geometry to *cis* or *trans*, while irradiation with a different wavelength switches the geometry to the other. *cis*- and *trans*-isomerisation of a moiety embedded in a macrocycle will have a profound effect on the structure of the system as a whole and the affinity with which it can bind a target protein. The most commonly utilised examples of this system are azobenzenes where the *trans*-isomer is sterically preferred. In these systems, irradiation with UV light switches the molecule to its *cis*-isomer and irradiation with visible light or heating reverts the molecule to the *trans*-state.

**Fig. 4:**
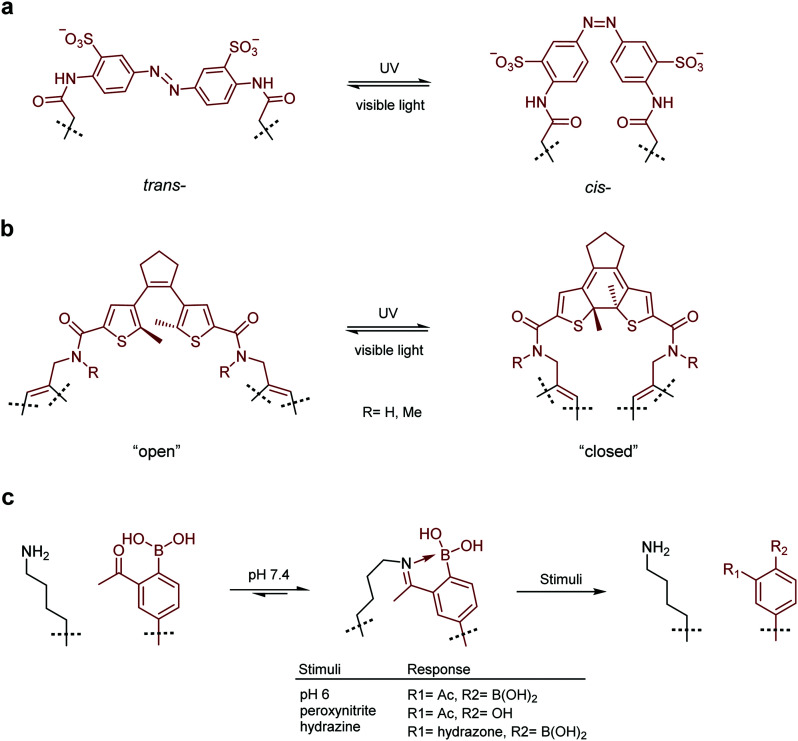
Switchable small molecule motifs for controlling peptide conformation. (a) Azobenzene-based linker for peptide cyclisation, UV light induces formation of the *cis*-isomer, which reverts to the *trans*-state when subjected to visible light. (b) Diarylethene-based cyclisation linker which adopts the “closed” form upon UV irradiation, switching back to the “open” form facilitated by visible light. (c) Iminoboronate-mediated peptide cyclisation, linkage readily cleaved by acidification (pH 6), oxidation (peroxynitrite) or treatment with α-nucleophiles (hydrazine). Small molecule components are highlighted in red.

In a prominent example, Nevola *et al.* created photo-switchable peptides to regulate clathrin-mediated endocytosis (CME) by targeting the beta-adaptin subunit of the AP2 complex, the most well characterised hub of the CME interactome.^132^ An azobenzene-based linkage (Fig. 4a) was used to alter the secondary structure of the macrocyclic peptides, and photo-switching of this moiety *in vitro* led to between 3- and 12-fold changes in affinity. The strategy was employed in a cell-based model where cell permeable analogues were found to photoregulate endocytosis of transferrin receptor protein in living cells.

Following this work, Wegner, Heinis and co-workers devised an *in vitro* evolution methodology to identify light-activatable macrocyclic peptide using the same azobenzene-based linker (Fig. 4a).^133^ Peptide binders of streptavidin were selected using phage-display. Crucially, the azobenzene motif was photo-switched to the *cis* conformation on the phage surface, prior to panning against the target protein. This facilitated the isolation of activatable peptides with an up to 3-fold binding selectivity over the *trans* form. More recently, Derda and coworkers extended this methodology by developing an azobenzene-based linchpin that could be used to create photoswitchable bicyclic peptides, though further work will be required to combine this methodology with phage display libraries.^134^

Whilst azobenzenes are by far the most heavily studied photo-switchable motifs, there are other groups which exhibit similar properties. Inspired by earlier studies,^135–137^ the Spring lab recently reported a photo-switchable hybrid molecule using a diarylethene (DAE, Fig. 4b) as the photo-switch.^138^ Incorporation of the DAE photo-switch into a known MDM2 binding sequence (pDI) maintained the low nanomolar binding affinity of the parent peptide in the “open” form, whilst reducing it up to 8-fold in the “closed” form.

Other methods for the development of switchable molecules rely on the use of chemical or environmental triggers^139^ such as pH,^140,141^ oxygen tension^142–144^ or the presence of an oxidising agent.^145,146^ These methods are now also being applied to peptides through the formation of peptide–small molecule hybrids. Gao and co-workers reported a reversible iminoboronate-mediated peptide cyclisation for tuning peptide activity.^147^ The imine product is stabilised by forming a dative bond with an adjacent boronic acid, resulting in robust stability at neutral pH against commonly seen biomolecules. Crucially, the iminoboronate linkage can be readily cleaved, with acidification to below pH 6.8, oxidation with peroxynitrite or addition of exogenous small α-nucleophilic molecules (Fig. 4c). The potential of this technique for biological applications was demonstrated by creating an iminoboronate-cyclised peptide which contained a potent and selective α_v_β_3_ integrin binding motif (RGDf, f = d-Phe). The binding interaction was successfully ‘switched-off’ in cells by lowering the cellular pH to induce peptide linearization, as the RGDf motif is only capable of binding integrin when placed in a suitable cyclic scaffold.

A well-established methodology related to photo-switchable molecules is photoaffinity crosslinking (or labelling). This approach has many advantages over other covalent methodologies. It is highly spatio-temporally resolved, crosslinking occurs rapidly and irreversibly, and unlike many other covalent approaches, it does not require a specific nucleophile for crosslinking. In the context of small molecules, it has been used for the identification of unknown binding partners, target validation,^148–150^ and fragment-based screening^151^ in both cell-based and purified enzyme settings. Once crosslinking has occurred the resulting species is usually characterised by proteomic mass spectrometry. This method allows investigation of both the target protein and the site of the protein which has been labelled. We have chosen to highlight a few articles from the most recent literature to demonstrate the different types of small molecule modification which are used with peptide hybrid photoaffinity probes. Other more general reviews which include photoaffinity probes are available.^152^

A recent example of a photo-crosslinking peptide probe was reported by Tate and co-workers during their investigation into the mode of action of the peptide Huwentoxin-IV and other cysteine knot peptides on voltage gated sodium (Nav) channels.^153^ To investigate the molecular mechanism of the potent modulation of Nav1.7, a library of aziridine (l-photomethionine) containing Huwentoxin-IV peptides was screened. Using proteomic mass spectrometry, a new model for binding of Huwentoxin-IV to the Nav1.7 channel was proposed.

A benzophenone functionality is a common alternative for photo-crosslinking. A recent example of this functionality being used to identify transient PPIs was demonstrated by An *et al.* in their investigation of phosphotyrosine binding modules such as the Src homology 2 (SH2) domain.^154^ By appending a benzophenone containing amino acid at the N-terminus and biotin at the C-terminus, a known SH2 domain was successfully captured and pulled-down. Screening for an optimised peptide sequence, using a combinatorial peptide ligand library subsequently identified a probe with which 24 SH2 domain proteins were captured.^155,156^ In addition, the technique identified six phosphotyrosine binding domains and five C2 domains which bind to phosphotyrosine. This study has laid the groundwork for a new mining strategy to identify putative binders of this PTM.

While the previous example incorporated the photo-crosslinking group on an amino acid side chain, inclusion of these motifs through the cyclising linker has also been described. Using their previously developed multi-functional dialkynyl cyclisation platform, Spring and co-workers were able to incorporate a benzophenone photoaffinity labelling motif into the linker. This allowed the development of peptides which preferentially cross-linked to MDM2 in the presence of BSA.^157^

Switchable and triggerable small molecule probes are an important class of compounds for chemical biology studies. The methods utilised to make these compounds are now being applied to peptide-based compounds through conjugation of small molecule fragments into peptide structures. This emerging class of peptide–small molecule hybrids is already showing potential in addressing complex questions and allowing the development of more potent peptide binders. Further developments in this field will undoubtedly continue to yield interesting and important molecules.

## Outlook

Peptides show immense promise as novel therapeutics or tools to investigate complex biological questions.^6,7,158^ A plethora of approaches are being developed to address the final hurdles to applying them more widely in the clinic and *in vivo*.^8,10^ In this review we highlight the potential peptide–small molecule hybrids provide both to tackle these limitations and to further extend peptide functionalities, including through enhancing binding potency, cellular stability or creating switchable tools.

The potential of hybrid approaches to extend peptide functionality is only just beginning to be realised and there is much scope for their expansion and general implementation. For example, conjugation of serum-binding small molecule tags to peptides is now a well-established approach to improve *in vivo* half-life.^89,93,159^ So far, however, the biological targets explored have all been extracellular, and it will be interesting to see the effect of these tags on cell permeability. A widely applicable small molecule tag that lengthens a peptide's *in vivo t*_1/2_ whilst retaining or even improving cell permeability would be highly coveted.

Similarly, switchable and triggerable small molecule probes are widely applied and immensely useful both for probing functionality and also as pro-drugs.^128,131^ Some of these modalities are already being included in peptide probes. Further incorporation of such small molecules into already validated bioactive peptides poses as a profitable opportunity to expand this class of compound and permit the investigation of more complex biological questions.

What is notable however, is that the majority of hybrid molecules are still tailored on a case by case basis. This often requires laborious rational design and multiple iterations of synthesis and testing to identify desirable compounds. Whilst effective, though slow, when applied to well characterised targets (where a crystal structure and small molecule or peptide binders are known) application to novel targets is more challenging. The lack of validated ‘one size fits all’ strategies remains a key hinderance in their wider use. With the growing ability to include these hybrid molecules in library screening strategies, however, the scope of targets available will increase exponentially, unleashing the full potential of this powerful class of molecules.

## Conflicts of interest

There are no conflicts of interest to declare.

## Supplementary Material
